# Cross-Country Differences in Basal and Stress-Induced Cortisol Secretion in Older Adults

**DOI:** 10.1371/journal.pone.0105968

**Published:** 2014-08-25

**Authors:** Juliana N. Souza-Talarico, Pierrich Plusquellec, Sonia J. Lupien, Alexandra Fiocco, Deborah Suchecki

**Affiliations:** 1 Department of Medical-Surgical Nursing, School of Nursing, Universidade de São Paulo, São Paulo, Brazil; 2 Department of Psychobiology, Universidade Federal de São Paulo, São Paulo, Brazil; 3 School of Psychoeducation, Université de Montréal, Montreal, Quebec, Canada; 4 Centre for Studies on Human Stress, Mental Health Institute of Montréal Research Center, Department of Psychiatry, Université de Montréal, Montréal, Quebec, Canada; 5 Department of Psychology, Ryerson University, Toronto, Ontario, Canada; Max Planck Institute of Psychiatry, Germany

## Abstract

**Objective:**

Several studies have emphasized the association between socioeconomic status (SES) and inadequate response of the biological stress system. However, other factors related to SES are rarely considered, such as cultural values, social norms, organization, language and communication skills, which raises the need to investigate cross-country differences in stress response. Although some studies have shown differences in cortisol levels between immigrants and natives, there is no cross-country evidence regarding cortisol levels in country-native elders. This is particularly important given the high prevalence of stress-related disorders across nations during aging. The current study examined basal diurnal and reactive cortisol levels in healthy older adults living in two different countries.

**Methods:**

Salivary cortisol of 260 older adults from Canada and Brazil were nalyzed. Diurnal cortisol was measured in saliva samples collected at home throughout two working days at awakening, 30 min after waking, 1400 h, 1600 h and before bedtime. Cortisol reactivity was assessed in response to the Trier Social Stress Test (TSST) in both populations.

**Results:**

Our results showed that even under similar health status, psychological and cognitive characteristics, Brazilian elders exhibited higher basal and stress-induced cortisol secretion compared to the Canadian participants.

**Conclusion:**

These findings suggest that country context may modulate cortisol secretion and could impact the population health.

## Introduction

Discrepancies in the prevalence of chronic diseases and stress-related disorders across nations have received widespread attention of scientists and international health organizations [1]–[3]. The World Health Organization (WHO) recently announced the global burden of chronic diseases, such as diabetes and cardiovascular disorders, showing that they are positively associated with 29% of deaths in people under the age of 60 in the low-middle income population compared to 13% in the high-income countries [2]. Similarly, according to the World Mental Health Survey Initiative, the highest prevalence of major depression varies from 10.4 in low to middle-income, to 8.3 in high-income countries [3]. Chronic exposure to high levels of stress has been associated to the development of various chronic illnesses including cardiovascular diseases, metabolic syndrome, central adiposity, diabetes as well as psychological disorders such as depression [4]–[8].

Differences in the prevalence of chronic diseases and stress-related disorders among countries raise the question of differences in the activity of the stress system between countries. While acute stress induces adaptive neuroendocrine and behavioral responses to challenging situations, repeated and prolonged stress can lead to dysfunction of the hypothalamic-pituitary-adrenal axis (HPA), the main neuroendocrine stress regulatory system. The central role of cortisol, the peripheral stress hormone in the pathway by which the environmental context can “get under the skin” damaging health and the course of aging, has been recently reviewed [7], [9]–[10].

Socioeconomic status (SES) has been the main environmental factor investigated, and findings show a significant relationship between low SES and high diurnal and evening salivary cortisol secretion [11]–[12]. In addition, individuals from low SES present lower levels of wake-up cortisol and flattened decline during the early part of the day than individuals from high SES [13]. However, in a given environment, there is much more than SES to characterize and differentiate people, including cultural, social and physical factors that may influence how people react to challenging situations [14]. For example, social support seeking, ethnicity as well as healthcare system have been described as important factors that differentiate nations, as to diseases, and even stress-related ones [15]–[18]. Interestingly, significant inverse associations between high social support and low cortisol levels as well as a direct correlation with better health outcomes have been demonstrated, indicating that the environment context, beyond SES, may play a role in the functioning of the stress system and therefore in the risk to develop stress-related disorders [19]–[21].

Despite these well-known environmental factors and differences between countries, most studies on the stress system have been performed in safe, rich, highly educated and developed country contexts that are not fully representative of most inhabitants around the world. With some exceptions, a few studies have been conducted to investigate the cortisol profile in non-developed country populations [22]–[25] and it is still difficult to determine whether the adaptive stress response is dysfunctional and/or whether there exists a relationship between this dysfunctional stress response and any chronic conditions in these populations.

Cross-country investigations are thus necessary to go forward into the investigation on the relationship between environmental context and stress response since they may provide important information regarding differences on patterns of cortisol secretion between populations from distinct social and cultural contexts. Moreover, cross-country findings may help scientific and health organizations to understand why and how people are more or less vulnerable to stress-related disorders in certain contexts [14].

Cross-country comparisons in the field of stress are mainly limited to self-administered perceived stress scales, and have provided various results: on the one hand, total work stress score appears not to differ between countries in, for example, nurse students [26]; on the other hand, country-specific differences in work stress are observed in physicians from different countries with the highest level in Germany, intermediate level in the US and lowest level among UK physicians [27]. In another study, authors found an almost threefold higher proportion of German physicians exhibiting high level of work-related stress [28]. Most of these studies address cross-country comparisons in relation with training or working conditions, and very few deal with stress in non-working population. We found only one wide range study with a total sample of 10,941 adolescents, in which no difference was detected across 20 countries in perceived stressfulness, although differences were found in the strategies these youth used to cope with stress [29]. Despite the relevant contribution provided by such psychometric measures, the multi-domain construct of stress also involves the interaction among environment stressors and biological stress activity [30], namely diurnal cortisol profile and cortisol reactivity to an acute stressor. In addition, such biological measures appear highly valuable in cross-country comparison studies since they prevent potential bias related to cultural interpretation of questionnaires.

Some studies have compared cortisol levels between adult immigrants and native inhabitants in the same country [13], [17], [31]–[33], but the risk of acculturation biases may underestimate the results. Therefore, best way to investigate differences in stress response between nations avoiding acculturation biases would be to compare the activity of the stress system between native individuals living in culturally, contextually and economically different countries. As far as we know, no such comparison has yet been done using stress hormones. Moreover, most cross-country comparisons in the field of stress have been performed in young and adult individuals, although older adults are especially vulnerable to the effects of chronic exposure to stress hormones, particularly regarding cognitive impairment and mental health disorders [9]. Given that the world population is growing older, it is relevant to understand how the environmental context can influence the stress response and, consequently, the quality of life and successful aging in different countries. Therefore, the aim of the present study was to examine basal and reactive cortisol levels in healthy older adults living in two culturally and economically different countries. We hypothesized that the country context would influence the HPA axis functioning leading to different patterns of baseline and stress-induced cortisol secretion in aging people. Given that no previous studies have been performed on this issue, we were not able to propose any reasonable hypothesis with regards to the direction of these differences.

## Methods

### Ethics Statement

The study was approved by the Ethical Committee of the Research Centre of the Montreal Mental Health University Institute, Montreal, Canada (#03/22) and by the Ethical Committee in Research of the Universidade Federal de São Paulo, São Paulo, Brazil (#428/2010). All participants signed an informed consent before the start of the study protocol.

### Participants and recruitment

Two hundred and sixty (52 men and 208 women) healthy older adults between 47 and 82 years of age (M = 61.9, SD = 7.4) with preserved global cognition, evaluated by the Mini Mental State Exam score (MMSE) (Mean = 28.4, SD = 2.4), were recruited from Montreal, Canada (n = 131) and from São Paulo, Brazil (n = 129). Montreal is a metropolitan city in Canada, a developed country with sustained growth and economic security, low birth rate, high life expectancy, high literacy level and trained workforce. São Paulo is the most populous city in Brazil, (almost three-fold more people than Montreal) a rapidly growing emerging country with a huge potential for additional growth, but significant social disparities in living conditions and education resulting in high unemployment and large inequalities in income and accessibility to health care [34]. Nonetheless, there are much more differences than socioeconomic characteristics between Canada and Brazil. Particularly, the inhabitants are exposed to different climates (temperature and seasonal variations), pollution level, crowding, noise, demographic factors, historical social roles, values, norms and organization, health service accessibility, language and communication skills. Sociodemographic, health indicators and climate characteristics of Canada and Brazil are shown in table 1.

**Table 1:** General sociodemographic, health indicators and climate characteristics of Canada and Brazil.

Characteristics	Canada	Brazil
***Sociodemographic characteristics***		
Total population (in millions)	34.017	194.946
Population over 60 years (%)	20	10
Annual growth rate (%)	1.0	1.1
Life expectancy at birth (years)	81	73
Life expectancy at age 60 (years)	24	21
Literacy rate among adults aged ≥15 years (%)	99	88.6
Crude death rate (per 1000 inhabitant)	7.1	6.3
Gross National Income per capita (US$)	38,310	11,000
Mean income among elders per capita (US$)	31,150	8,868
Population living below the national poverty line (%)	9.4	26
Health indicators		
***Health workforce***		
Physicians (per 10,000 inhabitant)	19.8	17.6
Nurses (per 10,000 inhabitant)	104.3	64.2
Hospital beds (per 10,000 inhabitant)	32	24
Mortality rate by cardiovascular disease and diabetes		
(ages 30–70 per 100,000 inhabitant)	82	248
***Climate patterns***		
Average min. and max. temperatures in °C* #	−12.4 min.	4.9 min.
	26.6 max	33.2 max.
Average daylight hours exposure per year*	12	16
Average daylight hours during summer and spring*	12.1	12.7

*Source*: World Health Organization 2012, The World Factbook, Central Intelligence Agency, 2008, *Instituto Nacional de Meteorologia* 2011, Metereological Service of Canada, 2011, Astronomical Applications Department of the U.S. Naval Observatory 2011.

* Data from Montreal and São Paulo.

# Minimum temperature during Wintertime and Maximum temperature during Summertime.

All participants were recruited using media advertisement (radio, television and internet) and were evaluated in their own country according to the same experimental protocol. Even though convenience sample is a relevant limitation to any generalization, our sample hold sociodemographic similarities with the Canadian and Brazilian general population as discussed forward in the limitation section.

Those who met the following criteria were excluded: neurological or psychiatric disorder, alcohol, smoking or drug abuse history in the last 10 years, use of antidepressant, benzodiazepines and synthetic glucocorticoids or steroid medication. All female participants from both countries were postmenopausal, not under hormone replacement therapy, which was an exclusion criterion. Cognitive and functional impairments were ruled out by administering the MMSE to Canadian and Brazilian participants [35].

### Diurnal salivary cortisol

Salivary samples were taken at the participants' homes with a cotton swab placed in the mouth for two minutes and stored in a plastic tube in the freezer. Detailed oral and written instructions were given to participants and included: to not practice exercise on the day of collection and to not eat or drink anything or brush the teeth one hour prior to saliva sampling. They were instructed to collect the saliva on two consecutive days at awakening, 30 minutes after awakening, 1400 h, 1600 h and at bedtime. Participants' compliance regarding timing of saliva sampling was assessed using a “daily sampling questionnaire” where individuals were asked to record the exact time of each saliva sampling. Compliant samples were considered only for the individuals who collected the first sample within 10 minutes of awakening and the second sample 30±7 minutes after awakening. In addition, compliance for the remaining three samples was defined as ±1 hour of the targeted time as recommended (1400 h, 1600 h and bedtime) [36]. Samples from participants who did not meet these criteria were excluded. Besides the diurnal profile of cortisol secretion, we analyzed the cortisol awakening response (CAR). CAR is a distinct feature of the HPA axis that responds to the endogenous stimulation of waking up by a peak occurring at 30–45 minutes after awakening [38].

### Stress reactivity

One week after home saliva sampling, a psychosocial stress was induced at Canadian and Brazilian settings using the TSST, a well-established and highly effective protocol that induces activation of the HPA axis [37]. The Brazilian scientist Dr. JN Souza-Talarico was trained at the Centre for Studies on Human Stress in Montreal by the team of Dr. S. Lupien on administration of the TSST in order to ensure that the team in each country used the exact same procedure for the stress task. The TSST consists of a 5 min public speech followed by a 5 min period of mental arithmetic task in front of a nonresponsive “behavioral experts” panel. Participants underwent the TSST in the afternoon between 1400 h and 1600 h. A total of six saliva samples for cortisol determination were obtained at −20 min (baseline), immediately before the TSST (−1 min), as well as immediately after (+1 min), and +15, +30, +45 min after the end of the TSST. To investigate the neuroendocrinology reaction of the body to the stress task we analyzed reactivity cortisol that represents the reactivity of the HPA axis to a stressful situation and the recovery cortisol that comprises the return of HPA axis activity to the condition prior to stress [37].

### Cortisol enzyme immunoassay

Canadian and Brazilian saliva samples were stored at −20°C until free cortisol levels were determined by a commercially available enzyme immunoassay kit (Salimetrics©, State College, PA, USA). The limit of detection for cortisol was 0.01 ug/dl, the intra- and interassay variability was 4.6% and 4.0%, respectively, in Canada, and 7.4% and 12.4% in Brazil (on a range of 0.1–10 ug/dl dose). Brazilian and Canadian research laboratories analyzed the salivary samples using the same enzyme immunoassay kit and followed the exact same Salimetrics recommendations. In addition, both laboratories were certified to use Salimetrics kits and the assay technique was previously validated.

### Statistical analysis

Cortisol levels were not normally distributed and therefore logarithm transformations were performed. Two-way analyses of variance (ANOVAs) for repeated measures were conducted to investigate possible effects of Time and Country (Canada×Brazil) and their interactions on diurnal and reactive cortisol levels. Greenhouse and Geisser (1959) method to correct the degrees of freedom was used when sphericity was not met. The CAR, as well as reactivity and recovery cortisol concentrations, were obtained by calculating the area under the curve (AUC) [56]. For CAR, AUC was calculated during the post-awakening period (from S1 awakening to S2 30 minutes post-awakening) [38]. The AUC from the pre-TSST to 15 min post-TSST comprises cortisol reactivity and the AUC from 15 to 45 min post-TSST represents cortisol recovery [56]. Age, education, retirement and income level were included in all reported analyses as covariates. *Post-hoc* analysis was performed using Student T-test. No sex effect was observed (p>0.1), so this variable was pooled across the analysis. Time of sampling was also compared between the groups and no significant difference was found (p>0.1), and in all cases the level of significance was established to *p*<0.05.

## Results

### Sociodemographic, physical health and psychological assessment

No difference was found between Canadian and Brazilian samples in regards to gender distribution, health status, psychological and cognitive assessment. However, Brazilian participants were significantly older. As expected, Brazilian participants had lower educational level and lower income level than Canadian participants, being mostly retired (Table 2).

**Table 2:** Sociodemographic characteristics, physical health and psychological assessment for Canadians and Brazilians.

	Country		
	Canadian	Brazilian	
Demographic	(n = 131)	(n = 129)	*p-value*
***Socio-demographic characteristics***			
Female: Male	101:30	107:22	0.279
Age (Mean ± SD)	58.1±3.9	65.7±8.0	<0.001
Educational level (Mean ± SD)	16.0±3.3	9.8±4.5	<0.001
Income Level (%)			
Low	41.9	30.5	
Medium	45.3	60.9	0.05
High	12.8	8.6	
Retired (%)	41.6	78.0	<0.001
***Physical health Status***			
Glucose (mmol/L)	5.2±0.5	5.6±0.9	0.109
Cholesterol (mmol/L)	5.3±0.8	5.1±0.9	0.512
Health risk for chronic diseases (% yes)^1^	45.8	54.2	0.308
***Psychological assessment***			
Depressive symptoms (Mean ± SD)^2^	1.6±1.7	2.0±1.5	0.212
Self-Esteem (Mean ± SD)^3^	24.8±4.7	25.1±1.8	0.392
MMSE (Mean ± SD)^4^	29.5±0.7	27.8±2.7	0.122

1 Anthropometric measures (Body Mass Index and Waist/Hip ratio according *WHO classification*);

2 Geriatric Depression Symptoms;

3 Rosenberg Scale of Self-Esteem;

4 Mini Mental State Examination.

### Differences on diurnal and reactive cortisol between countries

**Figure 1:**
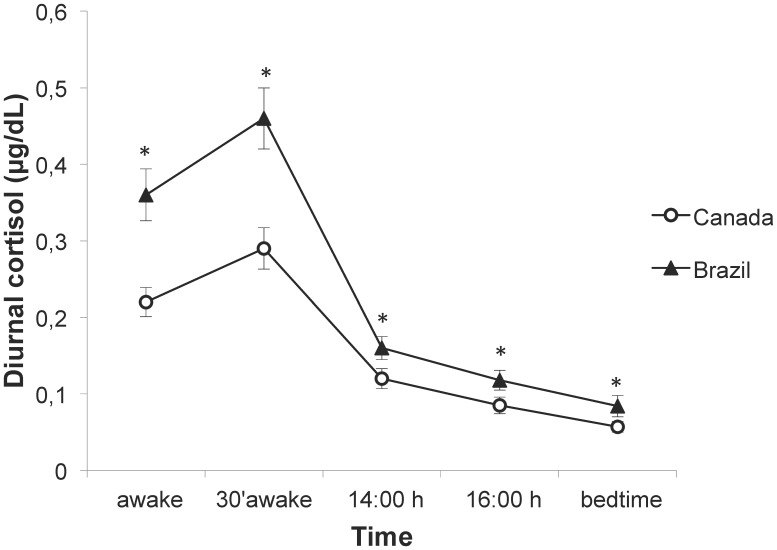
Diurnal cortisol concentration of older adults from each country. Brazilians (n = 126) showed higher diurnal cortisol concentration throughout the day than Canadians (n = 125). * p<0.001. Data is presented as mean ± s.e.m.

Controlling for age, education, retirement and income, ANOVAs for repeated measures showed a main effect of Country on diurnal cortisol levels (F(1,232) = 38.4, p<0.001), with salivary cortisol concentrations averaged across all time points differing between groups (p's<0.001; see Figure 1). No significant Time×Country interaction was observed (F(4,928) = 1.5, p = 0.207) demonstrating that cortisol profile over time did not differ between groups.

The cortisol awakening response (CAR) was compared between Brazil (mean: 0.11±0.22 µg/dL) and Canada (mean: 0.07±0.13 µg/dL), but no significant difference was observed (F(1,255) = 2.7; p = 0.101).

**Figure 2:**
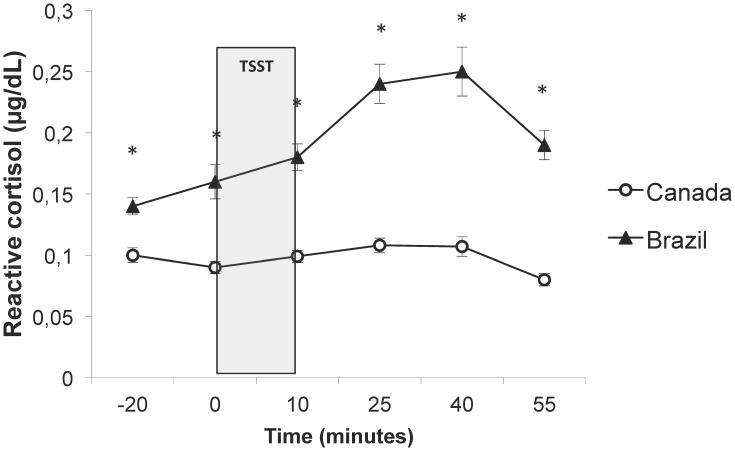
Reactive cortisol concentration of older adults from each country. Brazilians (n = 126) showed higher cortisol concentration before, during and after the TSST than Canadians (n = 125). * p<0.001. Data is presented as mean ± s.e.m. −20 min (baseline), 0 min  =  immediately before TSST, 10 min  =  immediately after TSST; 25 min; 40 min and 55 min after the end of the TSST.

Regarding reactive cortisol to TSST, controlling for age, education, income level and retirement, ANOVAs for repeated measures showed a significant Time×country Group interaction (F(3, 727) = 3.8, p = 0.009) and a main effect of country Group on reactive cortisol levels (F(1,230) = 52.8, p<0.001). These results suggest that groups differed in regard to cortisol secretion across all time points as well as in regard to profiles of cortisol concentration over time. Cortisol levels were indeed higher at all time points in Brazilian than in Canadian participants (p's<0.001; see Figure 2). A closer inspection of reactivity and recovery cortisol levels revealed significant differences between the countries, with higher reactivity but also higher recovery in Brazilian participants (mean reactivity: 0.09±0.22 µg/dL vs 0.02±0.06 µg/dL, F(1,252) = 12.1; p = 0.001; mean recovery: −0.05±0.09 µg/dL vs −0.03±0.05 µg/dL; F(1,252) = 5.7; p = 0.018).

There was no effect of age on either the within-subjects (Basal cortisol: F(4,928) = 1.435; p = 0.231; Reactive cortisol: F(5,1150) = 0.363; p = 0.790) or the between-subjects analyses (Basal cortisol: F(1,232) = 0.142; p = 0.706; Reactive cortisol: F(1,230) = 0.003; p = 0.956). Similarly, no effect of retirement was observed in the within-subjects (Basal cortisol: (4,928) = 0.520; p = 0.672; Reactive cortisol: F(5,1150) = 0.516; p = 0.681) or in the between-subjects analyses (Basal cortisol: F(1,232) = 0.239; p = 0.625; Reactive cortisol: F(1,230) = 0.340; p = 0.560).

## Discussion

In the present study, our hypothesis was that the country context might influence HPA axis functioning in older adults. We confirmed this hypothesis as we found that Brazilian participants showed higher diurnal and reactive cortisol concentrations than Canadians, even after controlling for potential confounding variables such as age, level of education, retirement and income.

Curiously, no statistical difference was found between Brazilians and Canadians regarding the cortisol awakening response. This result may indicate that the HPA axis response to the waking demands of the body remained unchanged across both countries [38].

Even though Canadians and Brazilians pertain to Western culture, living in urban settings, there are historical, social and organizational values differentiating them, which may influence the sensitivity and reactivity of the HPA axis to daily stressors, especially in aging people who are imprinted by their context. The effect of context has also been raised by Harrison (2001) who found that Australian aboriginal communities showed higher basal cortisol levels than Oxford city inhabitants [39]. Although this cultural difference could be attributed to the loss of traditional culture in most aboriginal groups, it could also be attributed to the lack of job opportunity and, therefore, the risk of boredom. Conversely, mean basal cortisol levels from the Tsimane Amazonian adult foragers were lower compared to the population of the CARDIA study, an important United States study [12], [25]. Even though abovementioned findings were not based on cross-country comparisons the authors stated that research should consider an integrative approach, including for example early social exposures, infectious disease loads, and nutritional and energetic status, to better understand these differences between studies performed in different countries. In our study, factors associated with higher level of retirement in the Brazilian sample may influence basal cortisol levels; thus, the difference in diurnal cortisol between Brazilians and Canadians should be considered in a broader perspective.

Regarding our results on cortisol reactivity following TSST, we found higher reactive cortisol to the TSST in Brazilians than in Canadians. This result could be explained by the fact that Brazilians are less familiar to public speech, which therefore would require more effort of verbalization than Canadians. Kim (2008) hypothesized that lower cortisol response to a stress verbal task in European Americans than in Asian Americans could be explained by a smaller effort for the former than for the latter to verbalize their thoughts and speech due to cultural norms [17]. Additionally, even though we have chosen to statistically control for the level of education in our analyses, it is reasonable to assume that the psychological demand to perform a mental arithmetic task could be higher in the Brazilian participants, leading to higher cortisol levels in response to the TSST. Supporting our interpretation, Fiocco et al. (2007) found higher change in cortisol baseline to post-TSST levels in middle-aged adults with low education when compared to middle-aged adults with high education level [40]. In their paper, Fiocco and colleagues argue that the TSST, as a verbal task, may represent a very stressful condition for middle-aged individuals with low level of education due to their low verbal capacity [40]. However, given that the main psychological determinants of the stress response behind the TSST are the uncontrollability and social-evaluative threat [57], differences in the sensibility/responsiveness to these characteristics may also explain the current findings. Thus, it could be possible that Brazilians, for culture and social reasons, are more sensitive to psychosocial stressors than Canadians. Unfortunately, we did not measure perceived stress after TSST to completely ascertain this hypothesis. Furthermore, participants of the Canadian sample appeared even to be no responsive to the task. Elderly women are known to be less reactive to the TSST than elderly men [41]. Combined with the higher level of education, the high percentage of women in our convenience sample may explain the low level of reactivity in the Canadian sample, and the observed difference between Brazilian and Canadian participants.

Another factor that could explain the differences in cortisol levels found between Brazilians and Canadians is related to the socioeconomic context. Even though the statistical analyses revealed that the differences were independent from income level, increased exposure to stressful daily events as well as fewer social and material resources are factors associated with lower SES [42]–[43]. Additionally, the psychosocial stress derived from relative deprivation of material resources in low SES groups has been proposed to be an important factor in the globally adverse impact of income inequality on health [44]. Our Brazilian and Canadian participants showed significant differences regarding income level and retirement, insofar as the average income per capita among elders in Canada is more than three-fold higher than in Brazil, where almost 7 millions of people live under poverty. Thus, it could be argued that unstable and unequal social conditions to which Brazilians are exposed to, may also explain the higher basal cortisol levels these individuals showed compared to the Canadians. Supporting this hypothesis, some authors have demonstrated high cortisol levels in low SES groups [11], [45]–[46]. However, the relationship between SES and basal cortisol levels is quite inconsistent in the current literature. While some authors found the opposite relationship [12]–[13], [47]–[49] others did not observe any association [50]–[53].

The last factor that could explain the observed cross-country differences in basal and reactive cortisol levels is related to environmental characteristics such as climate and light exposure. Seasonal variations, particularly long photoperiods, have been shown to stimulate higher cortisol levels [54]. Although data collection was done mainly during spring and summer in both countries, the amount of daylight hours could explain the differences we observed between Brazilian and Canadian participants since daylight hours are often longer in Brazil than in Canada (Astronomical Applications Department of the U.S. Naval Observatory). Nonetheless, although the differences in baseline cortisol levels could be explained by photoperiod, it is difficult to conceive that this factor could also impact on reactive cortisol levels measured within a laboratory setting in the two countries. Altogether, culture differences, SES, retirement, climate and light conditions and other factors not assessed in our study such as crowding, noise or pollution could be environmental variables that play a significant role in the activity of the stress system. Further studies should assess the impact of these factors in order to help us better understand the sensitivity of human populations to contextual, environmental, and cultural contexts.

### Limitations and future directions

In both countries, we recruited three times more women than men and this uneven sex distribution could explain some of our results. It is well known that women are more willing than men to participate in research studies [55] and this fact was observed in both countries. Given the low rate of men recruited, we were unable to detect any sex differences that might differ as a function of country for both basal and reactive cortisol levels. Furthermore, as expected, the groups were significantly different regarding sociodemographic characteristics such as age, education level, retirement and income. However, the statistical analysis showed that these variables did not affect either basal or reactive cortisol levels [40]. Moreover, it is important to highlight that sociodemographic differences between Brazil and Canada, including age and retirement, constitute peculiar characteristics of each country and that could (and should) not be eliminated in a cross-country approach. Additionally, the use of a convenience sample is a relevant limitation to any generalization regarding the differences observed. Even though our sample was not representative of the country population, its sociodemographic characteristics were not completely different from the general population of Canada and Brazil. Similarly to the current sample, there are more elderly women than men in Canada (74.8%) and Brazil (55.8%). Canadians are mostly distributed in the age range of 40 to 60, with high education level (Canada: 66.7% possess high school certificate, diploma or degree), with medium income level and within the 45–64 group the majority are not retired. In Brazil, the average age of elderly is 69 years old, 82.5% present up to 9 years of schooling, 72.2% pertain to the low and medium socioeconomic status and the majority is retired (57.95%) (Data from the Canada's National Statistical Agency and from the *Instituto Brasileiro de Geografia e Estatística*). The fact that our sample was similar but not representative of the entire population may attenuate, at least in part, the sampling bias. Finally, in future studies, we should expand our data collection to variables that may influence HPA functioning such as dietary habits, healthy behaviors, genetic combination, social support, perceived stress as well as past and daily life events.

### Conclusion

To the best of our knowledge, this is the first study showing that country contexts modulate cortisol secretion under basal and stress reactive conditions in older adults, even under similar health status, psychological and cognitive characteristics. Although it was not our objective to ascertain which country factor (culture, social context or environmental characteristics) influences the HPA axis functioning, the current results suggest that stress is likely to have a major impact on health in older adults from different environmental contexts. These results show that cross-country studies are necessary to analyze the health consequences of these differences across nations.
